# Abundance and antibiotic resistance of *Aeromonas* isolated from the water of three carp ponds

**DOI:** 10.1007/s11259-020-09768-x

**Published:** 2020-01-21

**Authors:** Marta Zdanowicz, Zbigniew Jan Mudryk, Piotr Perliński

**Affiliations:** Department of Experimental Biology, Institute of Biology and Earth Science, Pomeranian University in Słupsk, Arciszewskiego 22b, 76-200 Słupsk, Poland

**Keywords:** *Aeromonas* number, Antibiotic resistance, Pond

## Abstract

Abundance and antibiotic resistance of bacteria of the genus *Aeromonas* isolated from the water of three carp ponds were studied. The number of those bacteria differed between the studied ponds, sites and season. The results of the present study showed that planktonic *Aeromonas* inhabiting those ponds strongly differed in the resistance level to tested antibiotics. These microorganisms were the most resistant to amoxicillin, ampicillin, clindamycin and penicillin. However, all isolates *Aeromonas* were susceptible to gentamycin and streptomycin. Majority of bacterial strains were characterized by resistance to 4–6 of the 12 antibiotics tested. Bacterial resistance to antibiotics depended on their chemical structure. *Aeromonas* strains isolated from the studied ponds were the most resistant to β-lactam and lincosamides antibiotics, while the most susceptible to aminoglycosides, chloramphenicols and fluoroquinolones.

## Introduction

The genus *Aeromonas* taxonomically belongs to the class *Gammaproteobacteria*, order Aeromonadales and the family *Aeromonadaceae* (Dias et al. 2012). These bacteria are Gram-negative, facultative anaerobic, non-spore forming motile bacilli that inhabit marine and freshwater water basins (Piotrowska and Popowska 2014; Dar et al. 2016). According to Kozińska and Pękala (2010), and Hu et al. (2012) these bacteria are pathogenic to many aquatic animals and are particularly known as important fish opportunistic pathogens. These pathogens are responsible for diseases and mortality of different fish; mainly such species as carp, tilapia, rainbow trout, brown trout, eel, perch, catfish, goldfish and salmon (Hossain et al. 2014). Motile *Aeromonas* as pathogenic bacteria can kill up to 80–100% of fish within 1–2 weeks causing substantial economic losses, due to high mortality rates and worsened quality of produce in fishery farms, mainly for commercial carp farming (Orozova et al. 2010, Stratev et al. 2015; Mulyani et al. 2018). In particular such species as *Aeromonas hydrophila, A.caviae, A.sorbia, A. salmonicida, A. jandei, A.bestiarum* and *A*.*veronii* are typically associated with diseases and mortality in fish (Beaz-Hidalgo and Figueras 2013; Yu et al. 2015; Chenia 2016). These species are able to synthesize virulence and pathogenicity factors including hemolysins, aerolysin, leukocidin, cytotoxins, enterotoxins, proteases, gelatinase, elastase, lipase, phospholipases, DNase, and adhesin (Das et al. 2013; Yu et al. 2015). Several of these virulence factors have been identified in *Aeromonas* strains isolated from fish and many water ecosystems (Matyar et al. 2010; Desai and Desai 2014). These factors are the cause of many fish diseases, like external ulcerative lesions, fin rot, ocular ulceration, red sores, reddish head, rotting of the tails, anal region pale body colour, fin haemorrhagic, septicaemia, hemodiapedesis, anorexia, exophalmus and erythrodermatitis, revealed clear ascites, haemorrhage and destruction of sheathed tissues in spleen and renal tubular necrosis in the kidney, liver congestion, enlargement of spleen and kidney and enteritis resulting in major die-offs and fish kills (Yu et al. 2010; Hu et al. 2012; Rashid et al. 2013).

Several studies (Ozturk et al. 2007; Dias et al. 2012; Yu et al. 2015) suggest that different infections caused by *Aeromonas* are also closely associated with the change in environmental conditions and such factors as high stocking densities, overcrowding, a sudden change in water and air temperature, rough handling, abrasive handling, poor nutritional status, low dissolved oxygen, high levels of carbon dioxide, hypoxia, transfer of fish, mishandling, transportation which often involves traumatic events and stress factors to fish, non-bacterial pathogenic infections, poor water quality - mainly high levels of carbon dioxide and nitrate, organic pollution, and rough weather condition. All these environmental conditions decrease effectiveness of fish immune system which otherwise would clear up bacterial colonization and infection (Cabello 2006; Naylor and Burke 2005).

Intensive fish farming in recent years has resulted in growing problems of bacterial diseases, which led to a widespread antibiotic use for their treatment (Guz and Kozinska 2004; Daood 2012). Wide use of antibiotics to prevent and treat bacterial diseases and the application of subtherapeutic dose of antibiotics have inducted a global increase in the levels of antibiotic resistance among pathogenic bacteria in fish farming (Patil et al. 2016; Mulyani et al. 2018). As a result, the development of antibiotic resistance among aquatic bacterial pathogens will ultimately reduce the efficiency of antimicrobial agents used for treating and can favour the development of resistant bacteria in native fish species (Belèm-Costa and Cyrino 2006). Moreover, the increased antibiotic resistance confers bacterial pathogens an additional virulent feature, which generates increased mortality of fish in commercial farms (Daood 2012).

According to Dias et al. (2012) and Patil et al. (2016) *Aeromonas* strains are known as good indicator bacteria suitable for studying the incidence and development of antibiotic resistance in fish farms. Therefore, the aim of this study was to determine the number and investigate antibiotic resistance profiles among *Aeromonas* strains isolated from the water of carp (*Carpinus carpio)* three ponds.

## Material and methods

### Study area and sampling

This study was carried out in three carp ponds, fry, fingerling and adult located in Wiklino (North Poland); their characteristics was given in Table 1. A common feature of all these ponds is the cycle of filling and emptying the reservoirs with river water during the period of its excess or deficit used in traditional carp farming. Fish in the studied fish farm were adequately fed with the commercial pelleted feed.

**Table 1:** Values of selected morphometry and some physicochemical parameters of studied ponds.

Parameters		Fry pond	Fignerling pond	Adult pond
Pond type		artificial	artificial	natural
Area		0.5 ha	3 ha	3 ha
Average depth		0.7 m	0.8 m	0.9 m
Temperature	spring	17.2	17.3	16.8
	summer	22.0	22.7	20.3
	autumn	9.3	9.7	8.7
pH	spring	7.2	7.7	7.7
	summer	7.8	7.5	7.8
	autumn	7.8	7.7	7.8

**Fig. 1:**
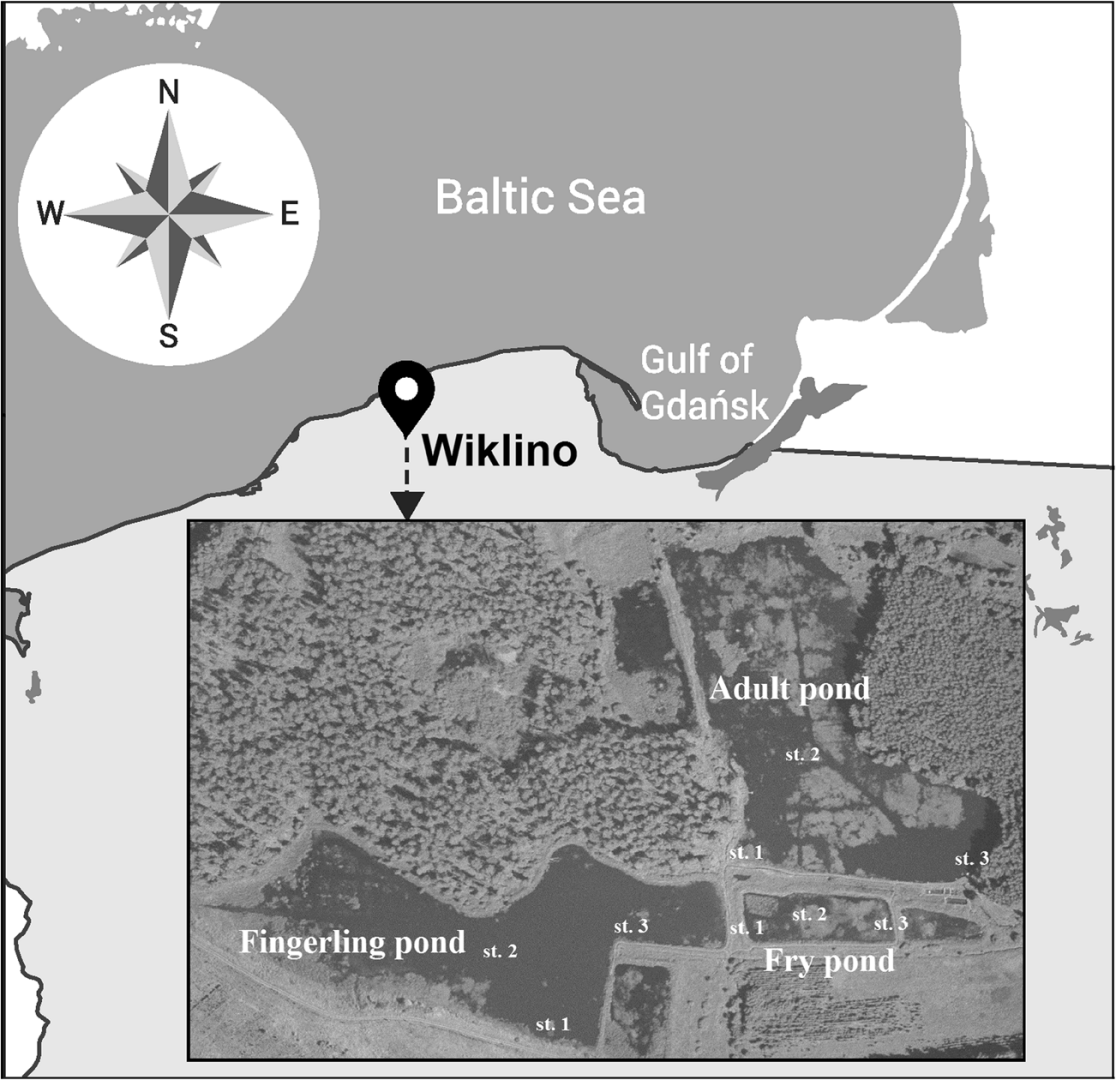
Map studied ponds where the sampling sites are located

Water samples were collected from each pond at three sites (Fig. 1) in the spring, summer and autumn seasons in 2013:

- Site 1 – located in the zone near the water inflow,
- Site 2 – located in the central part of the pond,
- Site 3 – located in the zone near the water outflow.

Water samples were collected from the depth of about 15 cm below the water surface directly into sterile glass bottles. Collected water samples were stored in an ice - box, where the temperature did not exceed **+**7 °C, and immediately transported to the laboratory. Microbiological assays as a rule were conducted within 4–6 h from the time of the sample collection.

### Determination of the abundance of Aeromonas bacteria

According to the procedures described by Mudryk et al. (2015) in order to determine *Aeromonas* abundance in the collected samples of water, these samples were diluted with sterile phosphate - buffered saline to reach final concentration ranging from 10^−1^ to 10^−3^. Diluted water samples were filtered through a 0.45 - μm pore size, 47 mm - diameter membrane filters (Whatman ME 25/31 ST). The filters were then aseptically transferred to the plates containing 10 ml of *Aeromonas* Isolation Agar (Biocorp) with ampicillin as a selective agent to reduce the growth of non-aeromonads (Jenkins and Taylor 1995). The plates were incubated at 37 °C for 48 h in portable incubator. After incubation, the plates presenting dark green, opaque with darker center colonies were determined according to Clark et al. (2003) as presumptive *Aeromonas*.. Number of *Aeromonas* was counted and results were calculated as colony forming units (CFU) per 1 ml of water. All presumptive *Aeromonas* bacterial isolates were than subject to various characterizations which eventually led to their identification. A series of morphological (shape, size, Gram character, flagellation) and conventional biochemical tests were performed to characterize the suspected *Aeromonas*. All strains which were Gram negative rods, positive for such enzymes as cytochrome oxidase, catalase and DNase, oxidative-fermentative (OF), acid and gas production from sugars (glucose, lactose, maltose, sucrose and manitol), methyl-red, H_2_S production, were according to Jeeva et al. (2013) and Rashid et al. (2013) identified as *Aeromonas*.

### Antibiotic resistance profiling of Aeromonas spp. isolates

A total of 106 *Aeromonas* spp. isolates were determined based on their profiles of antibiotic resistance according to the single disc diffusion method described in details by Mudryk et al. (2015). In order to determine antibiotic resistance, bacteria were multiplied on Mueller - Hinton (M - H) (Oxoid) agar slants at 20 °C for 24 h. Multiplied bacteria were washed off with 5 ml of sterile phosphate - buffered saline and the optical density culture was adjusted to 2.5 MacFarland standard units. Subsequently, 0.2 ml of bacterial suspension prepared in this way was introduced into dissolved M-H agar cooled to 40–45 °C. After mixing, the sample was poured onto Petri dishes and dried in a drier at 37 °C for 1 h. Paper discs impregnated with an antibiotic were then applied to the surface of the seeded medium with an automatic disk dispenser at distances no less than 2 cm. The blotting paper discs (φ13 mm) were manufactured by Oxoid Company. The dishes were kept at 4 °C for 1 h in order to allow antibiotic diffusion from the discs into the agar medium and then incubated at 20 °C for 24 h. The degree of resistance or sensitivity of the strains was determined on the basis of the measurements of lightened zones (in mm) around the disc and comparing them with the data given by the manufacturer instructions. The strains showing resistant or intermediate behaviour were subsumed under the category resistant. All others strains were classified as sensitive. The following twelve antibiotics (their codes and concentrations [μg disc] were given in parentheses), which are commonly used in clinical practice and in aquaculture were tested in antibiograms: amoxicillin (AX 25 μg), ampicillin (AM 10 μg), chloramphenicol (C 30 μg), ciprofloxacin (CIP 5 μg), clindamycin (CA 2 μg), erythromycin (E 15 μg), gentamycin (GN 10 μg), neomycin (N 30 μg), oxytetracycline (OT 30 μg), penicillin (P 10 μg), streptomycin (S 300 μg) and tetracycline (TE 30 μg) manufactured by Oxoid Company. The results were used to calculate the Antibiotic Resistance Index (ARI = no. of antibiotics to which the isolate was resistant / total no. of antibiotics tested) (Webster et al. 2004). The strain *Aeromonas hydrophila* ATCC 7966 was used as control for verification of the anti-bacterial effect of the disc on Muller-Hinton agar plates (Yucel et al. 2005).

All tested antibiotics according to their chemical structure were divided into seven groups: aminoglycosides (AMG) (gentamycin, neomycin, streptomycin), β-lactams (LA) (amoxicillin, ampicillin, penicillin), chloramphenicols (CHL) (chloramphenicol), lincosamides (LIN) (clindamycin), macrolides (MAC) (erythromycin), fluoroquinolones (FLU) (ciprofloxacin) and tetracyclines (TET) (tetracycline, oxytetracycline) (Reinthaler et al. 2003). Isolated *Aeromonas* strains were also analyzed for the multiple antibiotic resistance (MAR) according to Mudryk et al. (2015).

### Statistic analyses

Statistical tests (standard deviation-SD, coefficient of variation-CV, coefficient of dispersion-CD) used in this analysis were based on Velji and Albright (1986). Relationships among parameters within the whole data set were examined using Spearman’s rank correlation coefficient using Statistica software. The significance of differences between ponds, seasons and sites in level of number of *Aeromonas* was assessed using Kruskal - Wallis non-parametric equivalent of ANOVA, when mean values revealed a distribution other than normal.

## Results

The data on the abundance of bacteria representing the genus *Aeromonas* in water samples collected at the study sites of three carp ponds were given in Table 2. According to these data, *Aeromonas* were the most abundant in fingerling pond (mean: 2.96·10^2^ CFU/ml), while their number was the lowest in adult pond (1.93·10^2^ CFU/ml). The highest average number of these bacteria (3.11·10^2^ CFU/ml) was isolated from water samples collected in the zone near the water outflow (st.3) from the studied ponds, while the lowest (2.22·10^2^ CFU/ml) was recorded in the zone near the water inflow (st.1). In fry pond, *Aeromonas* bacteria were the most abundant at the sites 1 and 3 (2.89·10^2^ CFU/ml) and their number was the lowest (2.22·10^2^ CFU/ml) in the central part of the pond (st.2). In fingerling pond, the maximum number of bacteria of the genus *Aeromonas* was noted at the site 3 (4.22·10^2^ CFU/ml) located near the water outflow from the pond, while the minimum (1.78·10^2^ CFU/ml) at the site 1 near the water inflow. The maximum number of bacteria of the *Aeromonas* in adult pond was recorded at the site 3 (2.22·10^2^ CFU/ml) and the minimum (1.56·10^2^ CFU/ml) at the site 2.

**Table 2:** Occurrence and abundance of *Aeromonas* in water samples collected from studied carp ponds (data derived from the pooled data of all seasons)

Pond	Site	CFU (ml)	Range	SD	CV(%)	CD
fry	st. 1	289	0–733	390	135.0	526.5
	st. 2	222	0–600	329	148.1	487.1
	st. 3	289	200–400	102	35.3	36.0
	average	267				
fingerling	st. 1	178	133–267	76	42.8	32.5
	st. 2	289	67–533	234	81.0	189.5
	st. 3	422	200–667	234	55.4	129.7
	average	296				
adult	st. 1	200	67–400	176	88.0	154.9
	st. 2	156	0–467	269	172.9	465.2
	st. 3	222	0–533	278	125.1	347.8
	average	193				

**Fig. 2:**
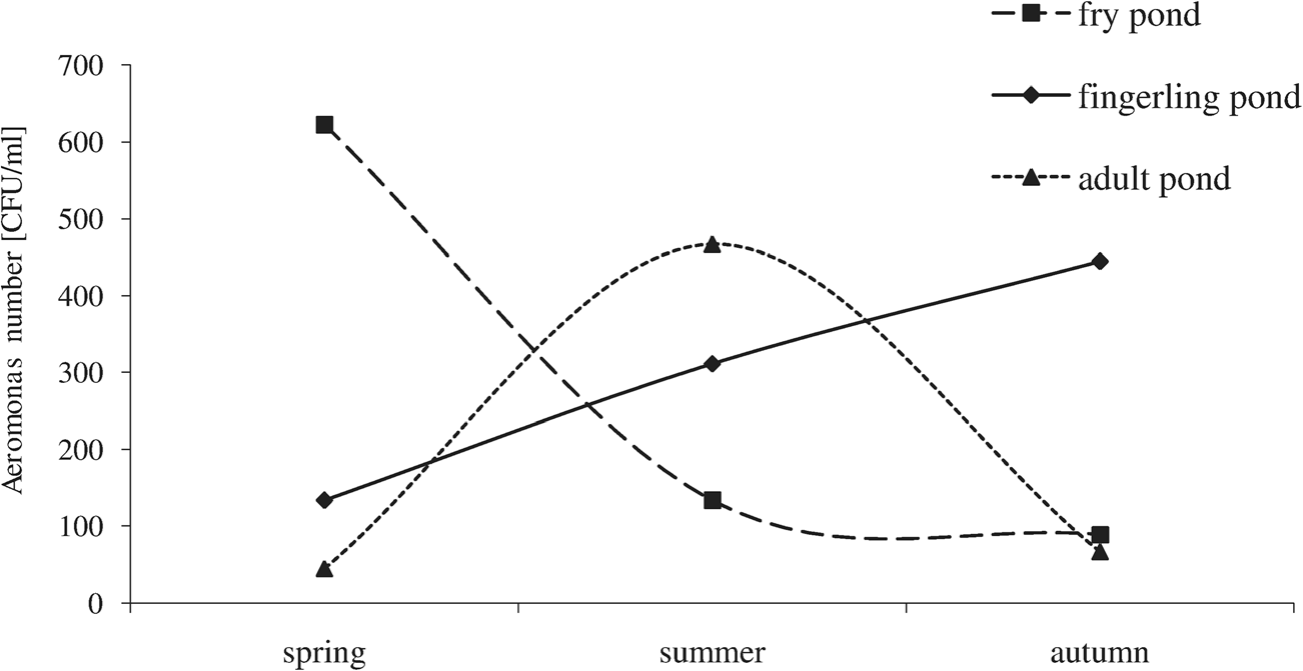
Seasonal dynamics change of *Aeromonas* bacteria number in water studied ponds (average from the pooled data of all sites and seasons)

The number of *Aeromonas* bacteria inhabiting studied carp ponds showed clear seasonal dynamics (Fig. 2). The highest average number of *Aeromonas* (3.03·10^2^ CFU/ml) was recorded in the studied ponds in summer and the lowest (2.00·10^2^ CFU/ml) during the autumn season. The maximum number of the studied bacterial group was noted in fry pond in spring, while during summer these bacteria were the most abundant in adult pond, and in the autumn season – in fingerling pond.

The data presented in Table 3 showed that *Aeromonas* isolates significantly differed in the level of resistance to twelve studied antibiotics commonly used in human medicine, veterinary and aquaculture. In all studied ponds the highest (96–99%) average percentage of all tested *Aeromonas* strains was resistant to amoxicillin, ampicillin, clindamycin and penicillin. About 60% of isolates was also resistant to erythromycin. On the other hand only 5–6% of tested strains were resistant to chloramphenicol and ciprofloxacin. However, all *Aeromonas* isolates were susceptible to gentamycin and streptomycin. The Antibiotic Resistance Index (ARI = 0.4–0.5) of planktonic *Aeromonas*. indicated that there was no difference in the level of antibiotic resistance between the studied fish ponds.

**Table 3:** Percentage of antibiotic resistance among *Aeromonas* isolated from water of three studied ponds

Antibiotics			Pond		
			fry	fingerling	adult
			Strain number		
	concentration	code	35	34	37
amoxicillin	25 μg	AX25	94.6	97.1	97.1
ampicillin	10 μg	AMP10	94.6	97.1	97.1
chloramphenicol	30 μg	C30	10.8	0.0	2.9
ciprofloxacin	5 μg	CIP5	10.8	8.8	0.0
clindamycin	2 μg	DA2	94.6	97.1	97.1
erythromycin	15 μg	E15	62.2	61.8	60.0
gentamicin	10 μg	CN10	0.0	0.0	0.0
neomycin	30 μg	N30	10.8	35.3	2.9
oxytetracycline	30 μg	OT30	29.7	32.4	11.4
penicillin G	10 μg	P10	100	97.1	100
streptomycin	300 μg	S300	0.0	0.0	0.0
tetracycline	30 μg	T30	27.0	32.4	8.6
ARI			0.4	0.5	0.4

**Fig. 3:**
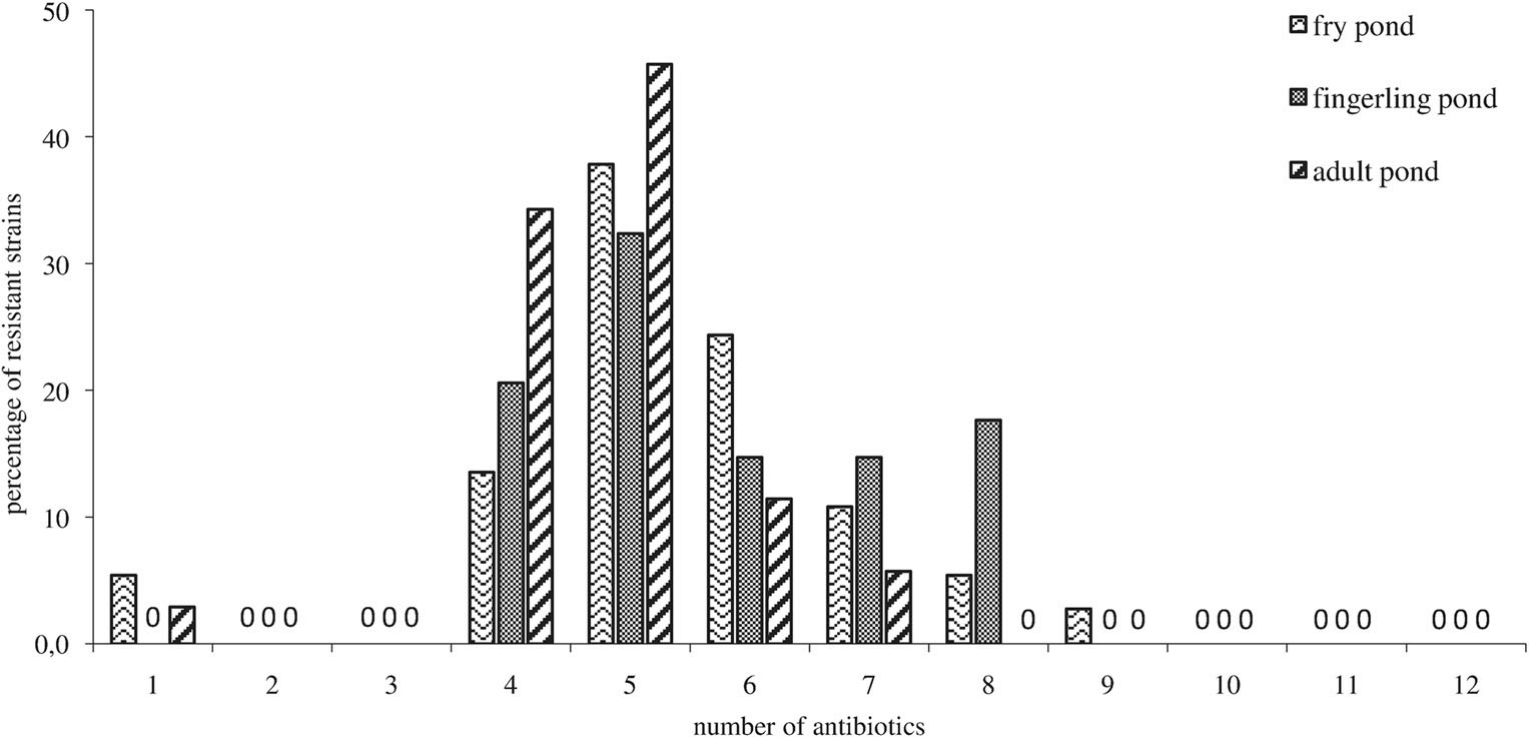
Percentage of resistance to 12 antibiotics of the *Aeromonas* strains isolated from three farmed carp ponds (data derived from the pooled data of all sites during the study period)

Isolated *Aeromonas* strains were also analysed for the multiple antibiotic resistance (MAR) (Fig. 3). From 17 up to 39% of planktonic *Aeromonas* strains showed resistance against four to six antibiotics and about 8–10% of tested strains were resistant to seven and eight antibiotics out of the twelve analysed drugs. None of bacteria inhabiting the studied ponds was found to be resistant to 1–3 and 11–12 of tested antibiotics.

**Fig. 4:**
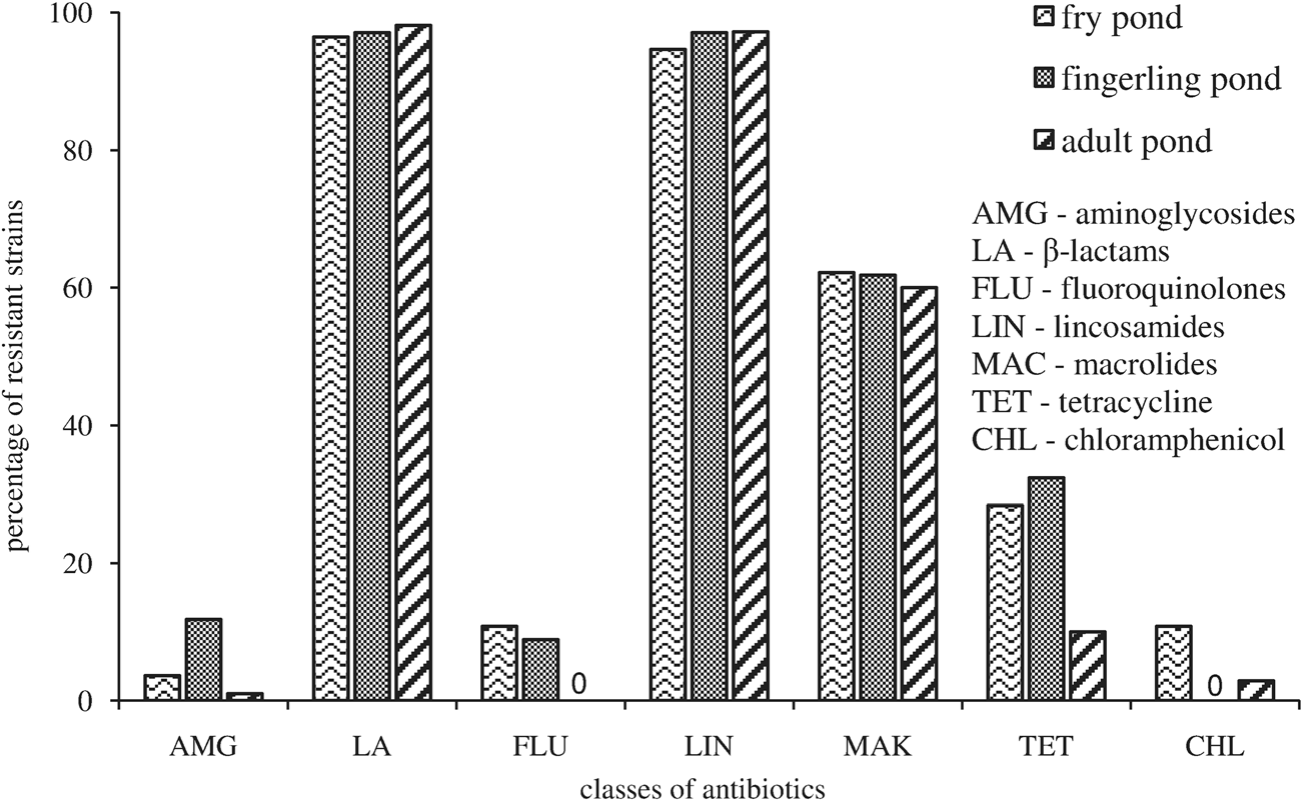
The resistance of studied bacteria with respect to their chemical structure (in percentage) (percentages derived from the pooled data of all sites and seasons)

The resistance level of *Aeromonas* to different classes of antibiotics is shown in Fig. 4. The results of present study showed that bacterial resistance depended on antibiotics’ chemical structure. *Aeromonas* isolated from the studied ponds were the most resistant to β - lactams and to lincosamides antibiotics. On other hand, isolated strains were the most susceptible to aminoglycosides, chloramphenicols and fluoroquinolones.

The relationships abundance of *Aeromonas* in studied fish ponds are given as the correlation matrix in Table 4. In water studied water basins noted positive (r = 0.82, *p* < 0.05) correlations between site 2 and site 3 and negative (r = −081, p < 0.05) correlations between summer and spring.

**Table 4:** Correlation matrix coefficient numbers of *Aeromonas* in the water fish ponds

Nonparametric Spearman’s correlation coefficients				
		fry	fingerling	adult
	fry			
ponds	fingerling	−0.36		
	adult	−0.21	0.33	
		spring	summer	autumn
	spring			
seasons	summer	−0.81**		
	autumn	−0.10	0.08	
		1	2	3
	1			
sites	2	0.65		
	3	0.59	0.82**	

Significance (p) is indicated by asterisks: ** *p* < 0.01.

By grouping the results by the ponds, seasons and sites Kruskal-Wallis test carried out to detect significant differences between the number of *Aeromonas* bacteria (Table 5). The number of *Aeromonas* non-significant differences between studied parameters were noted. Only significance difference (H = 18.947, *p* < 0.05) between ponds and seasons were observed.

**Table 5:** Analyses of the Kruskal - Wallis test in the numbers of *Aeromonas* in the water due to fish ponds, seasons and sites. Significance (p) is indicated by asterisks: * p < 0.05

Source of variation	H	p
ponds	1.703	ns
seasons	1.830	ns
sites	1.536	ns
ponds × seasons	18.947	*
ponds × sites	4.180	ns
seasons × sites	5.918	ns

Explanations:

H – the Kruskal – Wallis test

p – significance level

ns – non-significant

## Discussion

The genus *Aeromonas* along with *Pseudomonas* and *Vibrio* are the predominant microorganisms in ponds and these bacteria may be used as good biological indicators of water quality (Rippey and Cabelli 1989; Korzekwa et al. 2012). The number of *Aeromonas* bacteria inhabiting the water of three studied carp ponds varied from 1.93 to 2.96·10^2^ CFU/ml. This level number of *Aeromonas* was similar (2.4 ·10^2^ CFU/ml) to data reported by Leung et al. (1992) from aquaculture ponds at Aburn University and local fish farm in India (3.63·10^2^ CFU/ml) (Jha et al. 2008) but lower than the values noted by Gołaś et al. (2019) in aquaculture system farming of European grayling (11.0·10^3^ CFU/ml) and the number of *Aeromonas* (2.1–2.6·10^6^ CFU/ml) in river water of Lotcha (West Bengal,India) (Roy et al. 2013).

According to the obtained results, the number of *Aeromonas* bacteria inhabiting the studied ponds showed clear seasonal dynamics, which is consistent with the studies of Topić Popović et al. (2000) and Maalej et al. (2003). The maximum number of *Aeromonas* was recorded in the studied ponds in summer and the minimum in autumn. Summer maxima in the number of that taxonomic group may be the effect of relatively high temperatures at this time of year (Mudryk and Skórczewski 2007; Zdanowicz and Mudryk 2017). According to Cottrell and Kirchman (2000) temperature is a major abiotic factor influencing significantly the seasonal variation in the abundance of bacteria in aquatic ecosystems.

In recent years the number of antibiotic-resistant bacteria in aquaculture has increased dramatically in different parts of the world (Orozova et al. 2008). It is a consequence of the widespread and often uncontrolled use of antibiotics, prophylactically and therapeutically, against diseases, and also subtherapeutically as growth promoters for aquatic farm animals, mainly fish (Ozturk et al. 2007; Ramesh et al. 2010).

In the present study we showed that *Aeromonas* strains isolated from the water of three carp ponds were characterised by large differences in their resistance to particular antibiotics. Among all isolated strains the highest percentage (96–99%) was resistant to amoxicillin, ampicillin, clindamycin and penicillin. Significant percentage (70–100%) of *Aeromonas* strains showed resistance to β-lactam antibiotics, like amoxicillin, ampicillin and penicillin and similar results were reported by many researchers, for example, Yu et al. (2015) from the carp farm in Korea, Guz and Kozinska (2004) and Harnisz and Tucholski (2010) from carp ponds in Poland, and Yano et al. (2015) from inland ponds located in Bangkok (Thailand). According to Saavedra et al. (2004) the genus *Aeromonas* is considered naturally resistant to β - lactam antibiotics. Due to chemically unstable β - lactam ring in structure of β - lactam antibiotics, they are readily susceptible to bacterial hydrolysis by chromosomal β - lactamases produced by *Aeromonas* and are easily eliminated (Goňi-Urriza et al. 2000). In the present study, the high level of antibiotic resistance against amoxicillin, ampicillin and penicillin shown by many *Aeromonas* strains isolated from the water of the studied ponds indicated that the β - lactamase gene might be widely present in the gene pool of microbes in the studied aquatic environment (Lin et al. 2004).

In our study, *Aeromonas* strains inhabiting the water of three carp ponds were also characterized by high (96%) resistance to clindamycin. These results are comparable with the data obtained by Stratev et al. (2015) who noted that all *Aeromonas* strains isolated from rainbow trout were resistant to clindamycin and similar results reported also Mudryk et al. (2015) from marine water.

On the other hand, relatively low percentage (5–6%) of *Aeromonas* inhabiting the studied carp ponds showed resistance to ciprofloxacin and chloramphenicol. Similarly, only 0–10% of *Aeromonas* strains isolated by Hatha et al. (2005) in the farm of freshwater fish in India, Vivekanandhan et al. (2002) in fish collected from fish market (South India), Yano et al. (2015) in inland ponds located in Bangkok city were resistance to ciprofloxacin and chloramphenicol. According to Roberts (1993) chloramphenicol as well as ciprofloxacin are often used prophylactically in fish farms.

In the present study we observed that none of the isolated strains showed resistance to streptomycin and gentamycin. Our results on the sensitivity of *Aeromonas* strains to streptomycin and gentamycin are consistent with the results obtained by Radu et al. (2003) in seven fish farms of Merisian province (Turkey), Belèm-Costa and Cyrino (2006) from tilapia and pacu and Kanchan et al. (2016) from fish collected from local farm culture Kosumpisi District Maha Sarakham Province (Thailand) who showed that 100% of this taxonomic group of bacteria was susceptible to both antibiotics.

The present study showed multiple antibiotic resistance of planktonic *Aeromonas* strains inhabiting the studied ponds. The majority of these bacteria were resistant against four to six antibiotics of the twelve antibiotics used in this study. This means that they are capable of detoxification of those antibacterial organic compounds and indicates that most *Aeromonas* strains inhabiting the studied ponds originate from the high-risk sources of contamination, where antibiotics are often used (Orozova et al. 2008). Adaptive responses of bacterial communities to several antibiotics observed in the present study may have possible implications for the health of animals raised in the studied aquacultures (Rhodes et al. 2000; Orozova et al. 2008) and may reflect the history of antibiotic application (Hsu et al. 1992). Multiple antibiotic resistance in *Aeromonas* species inhabiting different water basins have been reported globally by many authors (Matyar et al. 2010; Yano et al. 2015; Deng et al. 2016). For this reason according to John and Hatha (2012) the development of multiple antibiotic resistance by different *Aeromonas* species, mainly such as *Aeromonas hydrophila, A. veronni, A.salmonicida, A.sorbia* and *A.caviae,* isolated from aquaculture environments (Igbinosa et al. 2012) in recent years has become a major problem in many parts of the world. The rapid increase in the number of resistant and multiresistant aquatic genus *Aeromonas* is due to the ability of these organisms to transfer antibiotic resistance by mobile genetic agents (plasmids, transposons, IS elements, gene cassettes, class 1 integrons) among bacterial populations by cell to cell contact (Dar et al. 2016; Patil et al. 2016; Piotrowska et al. 2017). Many of those mobile elements harbor multiple antimicrobial resistance determinants resulting in the propagation of antibiotic resistance in aquaculture environments (Patil et al. 2016).

Most classes of antibiotics that are used in medicine and veterinary are introduced into water basins (Ko et al. 2003; Lin et al. 2004). According to Reinthaler et al. (2003) and Mudryk (2005) bacterial resistance to antibiotics depends on their chemical structure. The occurrence of antibiotic resistance genes against different groups of antibiotics in the genus *Aeromonas* derived from aquacultures is widely known (Jacobs and Chenia 2007; McIntosh et al. 2008; Piotrowska and Popowska 2014); this is also confirmed by the results of the present study. *Aeromonas* strains isolated from the water of three studied ponds were the most resistant to β - lactam antibiotics. This class of antibiotics is the most widely used (approximately 50% of global antibiotic consumption) because they have low toxicity and are used to treat a broad range of infections (Livrmore 1996). β - lactam antibiotics inhibit the activity of enzymes participating in the biosynthesis of the bacterial cell wall by interrupting the trans peptidation process that links the peptidoglycan components of the bacterial wall to each other (Roberts 1998). The resistance of *Aeromonas* to β - lactam antibiotics is due to their ability to synthesize three extracellular enzymes: β - lactamase, acylase and penicillinase, which hydrolyse the amide bond of the β - lactam ring of β - lactam antibiotics and can also limit the permeability of cytoplasmic membranes to those antibiotics (Guz and Kozinska 2004; Saavedra et al. 2004). Thanks to the ability for the synthesis of these enzymes *Aeromonas* strains are capable of detoxifying those antimicrobial agents. The number of *Aeromonas* strains producing an extended spectrum of β -lactamases capable of hydrolysing β - lactam antibiotics is increasing, therefore resistance to β – lactams may become a serious problem all over the world (Schwartz et al. 2003).

Apart from β - lactam antibiotics, 96% *Aeromonas* strains isolated from the water of the studied ponds were resistant to lincosamide antibiotics. Lincosamides are one of the commonly used antibiotic classes in human and veterinary clinical practice, which occur in many water ecosystems (Andreozzi et al. 2006). High resistance of the studied taxonomic group of bacteria to these antibiotics is because lincosamides efficiently inhibit growth in Gram-positive bacteria, mainly staphylococcal and streptococcal forms, but have low activity against many Gram-negative bacteria, such as *Aeromonas* bacteria (Lüthje and Schwarz 2007). Resistance of *Aeromonas* strains isolated from the studied ponds to this class of antibiotics corresponds with the studies carried out in other water basins (Calamari et al. 2003; Andreozzi et al. 2006). According to Lüthje and Schwarz (2007) bacterial resistance to lincosamide antibiotics can be due to target site modification, active efflux mechanisms, mutations and enzymatic inactivation on the drugs. Our results also indicated that *Aeromona*s strains were able to follow at least one of the resistance mechanisms mentioned above, since such a high percentage of those organisms were resistant to lincosamide antibiotics.

Several studies (Ko et al. 2003; Orozova et al. 2008; Yu et al. 2015) reported that *Aeromonas* isolates are susceptible in vitro to aminoglycoside antibiotics. Also *Aeromonas* strains isolated from the water of the studied ponds apart from chloramphenicols and fluoroquinolones were the most susceptible to aminoglycoside antibiotics. This means that the studied genus of bacteria is not capable of actively detoxifying those antimicrobial agents. Aminoglycosides are a large and diverse class of antibiotics, which have bactericidal activity against some Gram - positive and many Gram - negative organisms (Ryu and Rando 2001). Aminoglycoside inhibition of bacterial cell growth occurs by inhibition of one or more of the biochemical steps involved in translation on the ribosome and disrupts the integrity of the bacterial cell membrane (Wright 2003).

## Conclusions

In conclusion, we pointed out that antibiotic resistance and multiple resistance to antibiotics of *Aeromonas* genus inhabiting the water of carp ponds still require explanation and prompt the need to evaluate their potential role in fish infections, in which antibiotic therapy would be required. Further studies are essential for better understanding of antibiotic resistance of *Aeromonas* in aquacultures particularly, where uncontrolled and extensive use of antibiotics may cause the frequent occurrence of multiple antibiotics resistance. The resistance of bacteria to antibiotics could be an important problem in the future, not only in fish health but also in public health as a result of the possible transmission of the antibiotic resistance to humans by fish carriers’ consumption.
